# Associations of bovine beta-casein and kappa-casein genotypes with genomic merit in Holstein Friesian cattle

**DOI:** 10.5194/aab-67-61-2024

**Published:** 2024-02-14

**Authors:** Sena Ardicli, Özgür Aldevir, Emrah Aksu, Kerem Kucuk, Ahmet Gümen

**Affiliations:** 1 Department of Genetics, Faculty of Veterinary Medicine, Bursa Uludağ University, Bursa, Türkiye; 2 Department of Obstetrics and Gynecology, Faculty of Veterinary Medicine, Bursa Uludağ University, Bursa, Türkiye; 3 Rumeli Farming, Kirklareli, Türkiye

## Abstract

The relationship between the index values used to evaluate the genomic value and essential markers such as casein genes provides important information at the herd level. Therefore, this study aimed to determine the association between casein gene genotypes and the indices of genetic merit in Holstein Friesian cattle. A total of 805 cows were genotyped using the Affymetrix^®^ Axiom^®^ array system. We used data composed of the total performance index (TPI) and net merit (NM) values as well as the predicted transmitting abilities (PTAs) indices, including milk, fat, fat percentage, protein, protein percentage, combined fat and protein (CFP), productive life (PL), somatic cell score (SCS), daughter pregnancy rate (DPR), livability (LV), udder composite (UDC), and feet–legs composite (FLC) of each animal. The statistical analysis consisted of a one-way analysis of variance (ANOVA) test followed by Tukey's test. The A2A2 and the AB genotypes were predominant in the *CSN2* and *CSN3* genes, respectively. The A2A2 animals were found to have higher TPI and NM values. Moreover, they exhibited higher PTA values for proteins, CFP, and PL. On the other hand, the A1A1 genotype was significantly associated with the highest UDC. Concerning the *CSN3* haplotypes, the BB animals had higher protein percentage and PL than alternative haplotypes. The AA and AB haplotypes were found to be significantly associated with the highest SCS and DPR values, respectively. In addition, the BE haplotype had the highest NM. Selection procedures focusing on casein genes in dairy cattle are becoming increasingly common worldwide, especially for A2 milk. However, herd-based dynamics are also fundamental to providing a desired genetic merit for the animals. This study may be valuable for further analyses regarding selection decisions using the breeding values of candidate animals in commercial dairy herds.

## 1 Introduction

Genomic selection is a form of marker-assisted selection (MAS) in which genetic markers covering the whole genome are used; hence, all quantitative trait loci (QTL) are in linkage disequilibrium with at least one marker (Goddard and Hayes, 2007). It allows the identification of animals with superior characteristics accurately.

Selection through the evaluation of phenotypic data and breeding values calculated based on pedigree records has been successful to a certain extent. The genetically supported selection programs developed with the widespread use of molecular techniques and the confirmation of the effects of discovered mutations and ​single-nucleotide polymorphisms (SNPs) on the phenotype have brought a new dimension to the situation (Goddard and Hayes, 2007; Hayes and Goddard, 2010). Studies on applications of MAS and, thus, its effectiveness both in the scientific community and the field have gradually increased. However, MAS has not been able to sufficiently ensure that the information in DNA is used intensively and effectively in selection programs. As a result, genetic improvement has been limited. Firstly, the traits of interest in livestock production were much more complex than expected. Thousands of genes determined these economically critical quantitative traits with minor effects on phenotype. Secondly, many genomic variants other than major genes were usually too small to be statistically significant and were ignored (Meuwissen et al., 2016). Genome-wide data provide researchers with more comprehensive information (Hayes and Goddard, 2010). This type of selection involves some basic application steps. Initially, a reference population is genotyped and recorded for the trait to estimate SNP effects. Further, selection candidates are genotyped, and the genomic estimated breeding value (GEBV) is calculated by combining their genotypes with the estimated impacts. The genomic selection approach does not require pedigree recording. Moreover, the selected animals are not necessarily trait-recorded (Meuwissen et al., 2016). These are essential constituents for the traditional best linear unbiased prediction (BLUP) estimated breeding value (EBV). Indeed, genomic selection can predict more accurate EBVs than is possible with pedigree and phenotypes alone (Goddard and Hayes, 2007).

In genomic selection, some indices can be used to assess genomic predictions effectively in order to provide more accuracy and achieve much quicker genetic improvement. In this context, the predicted transmitting abilities (PTAs) and total performance index (TPI) represent brief information about milk performance traits and health generated within comprehensive statistical estimations. The TPI and PTA are conducive values for statistical analysis because (1) they are highly processed data in which most nongenetic influences are excluded and (2) they represent the relative effectiveness of traits of interest (Kaminski et al., 2002). The PTA includes the deviation of the daughter's performance from the population mean adjusted for the mate's genetic merit and the grandsire and granddam (Rodriguez-Zas et al., 2002). The TPI combines the PTA values for protein, fat, somatic cell score (SCS), productive life (PL), daughter pregnancy rate (DPR), and daughter calving ease (Rector, 2009). Moreover, the TPI comprises linear composite indexes such as the udder composite (UDC) and feet–legs composite (FLC). The TPI is the gold standard in ranking worldwide Holstein genetics, serving as a rudder for the genetic direction of the breed (Holstein Association USA, 2024), and it ranks animals on their ability to transmit a balance of these traits. According to the Holstein Association USA (2024), the weighting of major categories consist of production (46 %), health and fertility (28 %), and conformation (26 %). Although the most significant emphasis is still placed on production, health and fertility parameters are gradually increasing in importance in the index. Undeniably, these parameters are indispensable for sustainable dairy cattle breeding. For instance, the PTA for cow livability (LV) reflects a cow's capacity to remain alive on the farm, whereas the productive life (PL) signifies the animal's ability to avoid mortality or culling on the farm (Wright and VanRaden, 2016). It is worth noting that the TPI and PTA are dynamic values that are continuously corrected by inputting new data of the bulls' progeny and relatives' performance (Kaminski et al., 2002).

*Bos taurus* autosomes 6 (BTA 6) is a famous chromosome in dairy cattle breeding because it harbors the casein locus, which contains four closely linked milk protein genes. Concerning milk production traits, it is one of the most studied chromosomes as well as the location of the significant QTL (de Koning, 2006). In addition to their relation to the protein content, caseins have been the subject of many scientific studies regarding milk intolerance, which has gained significant attention worldwide (Prasad and Kothari, 2022). For example, the mutation in the *CSN2* (also known as β -casein or beta-casein) gene [g.8101C > A (p.His67Pro)] is an alteration of CCT/CAT (codon alteration from cytosine–cytosine–thymine/cytosine–adenine–thymine) for the A1 and A2 alleles, respectively (Bell et al., 2006; Jiménez-Montenegro et al., 2022). This missense mutation causes the resulting peptide chain's in vitro and in vivo digestion patterns to change and release beta-casomorphin-7 (BCM7). BCM7 has opioid effects, and its adverse effects have been associated with many human diseases (Summer et al., 2020). A2 milk has been gradually gaining popularity; thus, many farms plan to convert their dairy cattle herds to the A2A2 genotype. Hence, genetic selection based on bovine casein variants is a scorching topic in animal breeding and genetics (Ardicli et al., 2023). As with *CSN2*, bovine *CSN3* (also known as κ -casein or kappa casein) haplotypes are also valuable genes in terms of both milk protein content and milk allergy.

Türkiye stands out as one of the frontrunner countries with respect to its substantial cattle population. The aggregate count of cattle is currently a notable 17 692 955. Among these, 8 606 709 constitute purebred animals, 7 764 430 are hybrids, and 1 321 816 belong to indigenous breeds. As of 2022, Türkiye's overall milk output amounts to 21 563 492 t. The predominant contributor to this figure is cattle-derived milk. In this respect, cow's milk accounts for 92.3 % of raw-milk output. The cornerstone of Türkiye's bovine resources is epitomized by the Holstein Friesian breed, which assumes a central position. Despite the country's noteworthy cattle presence, the per-animal milk production averages a relatively modest 3158 kg (Turkish Statistical Institute, 2023). This once again underscores the urgency for contemporary cattle breeding techniques supplemented by advanced molecular genetic methodologies in Türkiye.

Dairy cattle genetics is a dynamic industry, with breeding companies and breeders constantly facing new challenges (Kearney et al., 2005). The relationship between index values used for evaluating the genomic value and essential markers such as casein genes provides important information at the herd level. Therefore, this study aimed to determine the association between the bovine casein gene (*CSN2* and *CSN3*) genotypes and the indices of genetic merit in a sizable population of Holstein Friesian cows.

## 2 Materials and methods

### 2.1 Animals and genotyping

In this study, we used 805 purebred Holstein Friesian cows raised in a commercial herd in the Marmara region of Türkiye. All cows were subject to the same environmental conditions and were milked thrice daily. They were fed ad libitum with corn silage, alfalfa hay, oat hay, and pellet based on a total mixed ration formulated to meet NRC (2001). The farm's herd size totals 2700 cattle. The facility is equipped with a system consisting of 120 stall beds with a smooth surface, automatic scraper, and drain. Milking parlors facilitated the milking of cows, employing electronic apparatuses that autonomously documented milk quantities. An Allflex milking automation system (Allflex^®^, Livestock Intelligence) was employed to register the milk output of individual cows during each milking session. The farm consistently generates a daily milk production ranging between 39 and 40 t, with each individual animal contributing an average yield of 40–43 L d ${}^{-1}$ . The locale where the farm is situated experiences mean temperatures of 12.50 ${}^{\circ }$ C during spring, 23.77 ${}^{\circ }$ C throughout summer, 14.67 ${}^{\circ }$ C in autumn, and 4.27 ${}^{\circ }$ C during winter. The average annual precipitation in this area amounts to 585.6 mm.

Ear tissue samples from each cow were obtained. We performed the DNA isolation using Genetic Visions Rapid DNA preps with K buffer (Genetic Visions-ST, Middleton, USA). Next, we assessed the concentration range (ng µ L) and the absorbance ratio of 260 / 280 using a NanoDrop spectrometer (NanoDrop 2000c, Thermo Scientific, Wilmington, DE, USA). We genotyped all of the animals using the Affymetrix^®^ Axiom^®^ array (Affymetrix, Santa Clara, Ca, USA) incorporated into a custom 70 K high-density SNP array in a GeneTitan^®^ multi-channel platform (Affymetrix). Ultimate data were analyzed using the Axiom^®^ Genotyping Solution Data Analysis Guide (Rev4). The genotypes were also evaluated based on the polymorphisms at the bovine *CSN2* (ENSBTAG00000002632.6) and *CSN3* (ENSBTAG00000039787.3) genes, respectively. The methods described here have complied with all of the relevant national regulations and institutional policies for animal care and use. The study was approved by the Bursa Uludağ University Local Ethics Committee for Animal Research (approval no. 2022–15/02).

### 2.2 Evaluation of genomic data

Each cow's genomic PTA values with respect to the milk yield, protein, protein percentage, fat, fat percentage, combined fat and protein (CFP), PL, SCS, and DPR were evaluated based on the Vision + 20™ test (Genetic Visions-ST, Middleton, USA). The composite indexes, including UDC and FLC, were assessed. We also investigated TPI and net merit (NM) data based on the corresponding genotypes in the studied population. Table S1 in the Supplement shows the descriptive data on genetic merit values (PTAs, TPI, and NM) of the studied Holstein Friesian population.

### 2.3 Genetic variation in the casein genes

We determined the genotypic and allelic frequencies based on the suggestions by Falconer and Mackay (1996). Next, we tested the Hardy–Weinberg equilibrium (HWE) using a chi-square ( $\chi^{2}$ ) goodness-of-fit test. Considering the *CSN3*, HWE testing for multiple alleles was performed as described by Louis and Dempster (1987). Population genetic parameters, including heterozygosity (He), effective allele numbers (Ne), and the polymorphism information content (PIC), were calculated according to Nei and Roychoudhury (1974) and Botstein et al. (1980). The estimation of the Shannon–Weaver diversity index ( $H^{\prime }$ ) is as follows:

$$H^{\prime }=-\sum_{n=1}^{n}P_{i}^{2}\mathrm{ln}P_{i},$$

where $P_{i}$ is the proportion of each allele in the population and ln⁡ is the natural logarithm.

### 2.4 Statistical analysis

We used GraphPad Prism 9 (GraphPad Software, La Jolla, USA) as the statistical software in this work. The normality of the data was evaluated using the Anderson–Darling test. Comparison of the *CSN2* and *CSN3* genotypes based on the genetic merit values of the cows was performed using the one-way analysis of variance (ANOVA). We used Tukey's multiple-comparison test as a post hoc comparison.

## 3 Results

### 3.1 Genetic variability in the *CSN2* and *CSN3* genes

Table 1 presents the genotypic distribution of the *CSN2* and *CSN3* genes. It also shows the HWE test results and population genetics parameters. The minor allele frequencies for the *CSN2* and *CSN3* markers were 0.31 (A1) and 0.14 (E), respectively. The *CSN3* marker showed higher He and PIC values than *CSN2*. Ne approached 1.80 and 2.60 in the *CSN2* and *CSN3* markers, respectively. The genotypic distribution in *CSN2* was consistent with the HWE ( P>0.05 ), whereas *CSN3* showed a deviation from the equilibrium ( P<0.001 ). Regarding the biodiversity status, *CSN3* exhibited a higher Shannon–Weaver index value than *CSN2* (Table 1).

**Table 1:** Genotype and allele frequencies, population genetics parameters, and genetic diversity indices in bovine *CSN2* and *CSN3* genes concerning A1/A2 and A/B/E alleles, respectively ( n=805 ).

Locus	*CSN2*				*CSN3*					
Genotypes	A1A1	A1A2		A2A2	AA	AB	AE	BB	BE	EE
n	74	359		372	159	285	85	139	125	12
Genotype frequency (%)	9.19	44.60		46.21	19.75	35.40	10.56	17.27	15.53	1.49
Alleles	A1		A2		A		B		E	
Allele frequency	0.31		0.69		0.43		0.43		0.14	
Heterozygosity (He)	0.4278				0.6106					
Number of effective alleles (Ne)	1.7476				2.5681					
Polymorphism information content (PIC)	0.3363				0.5277					
HWE test ${}^{*}$	P>0.05				P<0.0001					
Shannon–Weaver diversity index ( $H^{\prime }$ )	0.9363				1.5810					

*CSN2*: beta-casein; *CSN3*: kappa-casein; n : number of experimental cows; HWE: Hardy–Weinberg equilibrium. ${}^{*}$ P>0.05 : consistent with HWE.

### 3.2 Relationship between the genetic markers and genetic merit of the cows

Table 2 shows the results of the statistical analysis based on the bovine *CSN2* genotypes in relation to the genetic merit of the animals. Animals with the A2A2 genotype had higher PTA values for protein ( P<0.05 ), CFP ( P<0.05 ), and PL ( P<0.01 ) than those with the A1A1 and A1A2 genotypes. Cows characterized with the highest SCS value were the heterozygotes ( P<0.05 ). The A2A2-genotyped cows exhibited significantly higher TPI ( P<0.01 ) and NM values ( P<0.001 ).

**Table 2:** The means and standard deviations for the association between the bovine *CSN2* genotypes and genetic merit in the studied Holstein Friesian cattle ( n=805 ).

Parameter	Genotypes			P value
	A1A1	A1A2	A2A2	
Milk yield	698.80 ± 72.90	680.60 ± 44.60	802.10 ± 51.70	0.075
Fat	33.46 ± 2.63	30.87 ± 1.61	35.47 ± 1.86	0.051
Fat %	0.022 ± 0.011	0.017 ± 0.007	0.016 ± 0.008	0.898
Protein	24.37 ± 1.74 ${}^{\mathrm{ab}}$	23.45 ± 1.07 ${}^{b}$	26.93 ± 1.24 ${}^{a}$	0.025
Protein %	0.007 ± 0.004	0.006 ± 0.003	0.005 ± 0.003	0.905
CFP	57.84 ± 3.92 ${}^{\mathrm{ab}}$	54.32 ± 2.40 ${}^{b}$	62.39 ± 2.78 ${}^{a}$	0.018
PL	2.67 ± 0.18 ${}^{\mathrm{ab}}$	2.51 ± 0.11 ${}^{b}$	2.98 ± 0.131 ${}^{a}$	0.002
SCS	2.91 ± 0.02 ${}^{\mathrm{ab}}$	2.93 ± 0.01 ${}^{a}$	2.91 ± 0.01 ${}^{b}$	0.026
DPR	-0.45 ± 0.18	-0.23 ± 0.11	-0.37 ± 0.13	0.358
LV	0.53 ± 0.21	0.45 ± 0.12	0.49 ± 0.14	0.917
UDC	0.65 ± 0.13 ${}^{a}$	0.23 ± 0.08 ${}^{b}$	0.27 ± 0.09 ${}^{b}$	0.020
FLC	0.27 ± 0.08	0.07 ± 0.05	0.16 ± 0.05	0.043*
TPI	2326.80 ± 21.30 ${}^{\mathrm{ab}}$	2331.70 ± 13.00 ${}^{b}$	2379.60 ± 15.00 ${}^{a}$	0.009
NM	375.60 ± 21.30 ${}^{\mathrm{ab}}$	369.50 ± 13.00 ${}^{b}$	432.90 ± 15.10 ${}^{a}$	0.000

CFP: combined fat and protein; PL: productive life; SCS: somatic cell score; DPR: daughter pregnancy rate; LV: livability; UDC: udder composite; FLC: feet–legs composite; TPI: total performance index; NM: net merit. ${}^{*}$ not substantiated in Tukey's test. ${}^{a,b}$ Means with different superscripts are significantly different.

**Table 3:** The means and standard deviations for the association between the bovine *CSN3* haplotypes and genetic merit in the studied Holstein Friesian cattle ( n=805 ).

Parameter	Haplotypes						P value
	AA	AB	AE	BB	BE	EE	
Milk yield	638.40 ± 57.60	662.20 ± 46.40	646.60 ± 66.10	728.00 ± 57.50	820.80 ± 60.50	867.00 ± 175.00	0.144
Fat	30.36 ± 2.08	33.58 ± 1.67	28.91 ± 2.38	35.05 ± 2.07	37.15 ± 2.18	34.55 ± 6.32	0.050
Fat %	0.019 ± 0.009	0.028 ± 0.007	0.014 ± 0.009	0.024 ± 0.009	0.018 ± 0.009	0.006 ± 0.026	0.823
Protein	23.25 ± 1.38 ${}^{\mathrm{ab}}$	25.68 ± 1.11 ${}^{\mathrm{ab}}$	22.77 ± 1.58 ${}^{b}$	28.00 ± 1.38 ${}^{\mathrm{ab}}$	28.60 ± 1.45 ${}^{a}$	21.21 ± 4.20 ${}^{\mathrm{ab}}$	0.004
Protein %	0.009 ± 0.003 ${}^{a}$	0.017 ± 0.003 ${}^{a}$	0.008 ± 0.004 ${}^{\mathrm{ab}}$	0.017 ± 0.003 ${}^{a}$	0.009 ± 0.004 ${}^{a}$	-0.023 ± 0.010 ${}^{b}$	0.004
CFP	53.61 ± 3.10 ${}^{\mathrm{ab}}$	59.26 ± 2.50 ${}^{\mathrm{ab}}$	51.67 ± 3.55 ${}^{b}$	63.05 ± 3.09 ${}^{\mathrm{ab}}$	65.75 ± 3.26 ${}^{a}$	55.76 ± 9.43 ${}^{\mathrm{ab}}$	0.007
PL	2.39 ± 0.15 ${}^{b}$	2.67 ± 0.12 ${}^{\mathrm{ab}}$	2.96 ± 0.17 ${}^{\mathrm{ab}}$	3.04 ± 0.15 ${}^{a}$	2.89 ± 0.15 ${}^{\mathrm{ab}}$	2.41 ± 0.44 ${}^{\mathrm{ab}}$	0.006
SCS	2.94 ± 0.01 ${}^{a}$	2.92 ± 0.01 ${}^{\mathrm{ab}}$	2.89 ± 0.01 ${}^{\mathrm{ab}}$	2.89 ± 0.02 ${}^{b}$	2.89 ± 0.01 ${}^{b}$	2.96 ± 0.04 ${}^{\mathrm{ab}}$	0.001
DPR	-0.08 ± 0.14 ${}^{\mathrm{ab}}$	-0.06 ± 0.11 ${}^{a}$	-0.02 ± 0.16 ${}^{\mathrm{ab}}$	-0.12 ± 0.14 ${}^{\mathrm{ab}}$	-0.57 ± 0.15 ${}^{b}$	-1.23 ± 0.42 ${}^{\mathrm{ab}}$	0.008
LV	0.44 ± 0.166	0.62 ± 0.13	0.97 ± 0.18	0.52 ± 0.16	0.39 ± 0.17	-0.01 ± 0.46	0.087
UDC	0.31 ± 0.09	0.34 ± 0.08	0.39 ± 0.12	0.52 ± 0.09	0.52 ± 0.10	0.19 ± 0.30	0.279
FLC	0.09 ± 0.06	0.06 ± 0.05	0.24 ± 0.07	0.08 ± 0.06	0.26 ± 0.06	0.25 ± 0.18	0.160
TPI	2335.70 ± 17.30	2363.90 ± 13.70	2335.30 ± 19.40	2375.70 ± 16.60	2387.30 ± 17.60	2278.50 ± 50.00	0.055
NM	357.10 ± 16.80 ${}^{c}$	397.90 ± 13.50 ${}^{\mathrm{abc}}$	366.30 ± 19.30 ${}^{\mathrm{bc}}$	425.70 ± 16.80 ${}^{\mathrm{ab}}$	442.50 ± 17.70 ${}^{a}$	366.60 ± 51.10 ${}^{\mathrm{abc}}$	0.001

CFP: combined fat and protein; PL: productive life; SCS: somatic cell score; DPR: daughter pregnancy rate; LV: livability; UDC: udder composite; FLC: feet–legs composite; TPI: total performance index; NM: net merit. ${}^{a,b,c}$ Means with different superscripts are significantly different.

Concerning the *CSN3* haplotypes, the results of the ANOVA are shown in Table 3. The BE haplotype exhibited the highest PTA values for protein, protein percentage, and CFP among the haplotype groups ( P<0.01 ). This haplotype was also associated with the highest NM value ( P<0.001 ). Further, we observed significant differences in the PL ( P<0.01 ), SCS ( P<0.001 ), and DPR ( P<0.01 ); for these values, the highest values were the BB, AA, and AB haplotype carriers, respectively.

## 4 Discussion

Genomic selection allows for the accurate identification of phenotypically superior breeders and provides flexibility to breeders based on their targeted production. The trend in recent years has been to focus on health and longevity rather than milk production traits, which approach the genetic limit, especially in Holstein Friesian breeding. The propensity for selection conditioned on certain specific genomic regions or mutations may result in the herd being converted without sufficient attention to many essential traits. Casein genes, mainly bovine beta-casein, are among the best and current examples of this situation in cattle breeding. The demand for the consumption of beta-casein A2 milk is increasing worldwide, and producing this exceptional product is highly preferable among breeders and consumers. This market potential often results in breeders and sizable farms focusing directly on the A2A2 genotype and effectively converting the herd to A2. Although it is a topic of debate, the production of A1-free milk is encouraged in many countries, such as the USA, New Zealand, and the United Kingdom, because of its health benefits compared with conventional milk (Giglioti et al., 2020; Miluchová et al., 2023; Summer et al., 2020). As Kearney et al. (2005) suggested, sire selection decisions by commercial dairy farmers are substantially more complicated when information is available for specific genetic loci. In selection programs based on a particular genomic variant, the genomic merits of the selected candidates are of great importance for the herd's future. For instance, the genomic merit of the A2A2 breeders is crucial information with respect to the herd's genetics. Although A2 conversion may seem easy on the surface, it requires a complex genetic evaluation in association with consideration of the initial allele frequencies, herd size, replacement rate, involuntary culling rate, and age of the animals. The final decision regarding yearlings also varies depending on the availability of A2 semen and sex-selected semen (Mencarini, 2013).

**Figure 1:**
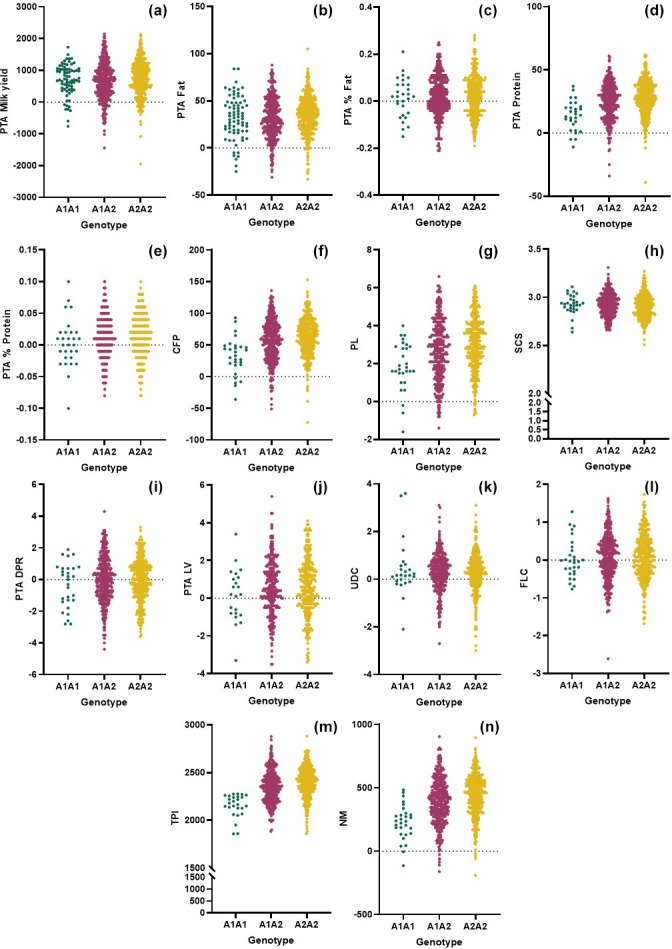
Comparison of the genomic merit values (PTAs) based on the *CSN2* genotypes with respect to the **(a)** milk yield, **(b)** fat yield, **(c)** fat content, **(d)** protein, **(e)** protein content, **(f)** combined fat and protein (CFP), **(g)** productive life (PL) **(h)** somatic cell score (SCS), **(i)** daughter pregnancy rate (DPR), **(j)** livability (LV), **(k)** udder composite (UDC), **(l)** feet–legs composite (FLC), **(m)** total performance index (TPI), and **(n)** net merit (NM).

**Figure 2:**
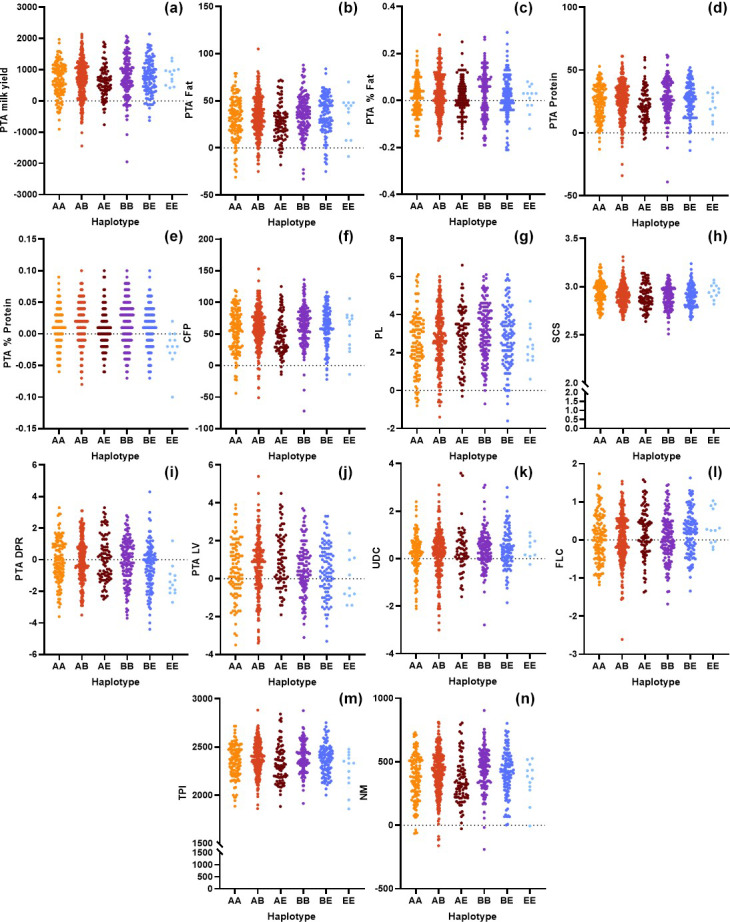
Comparison of the genomic merit values (PTAs) based on the *CSN3* genotypes with respect to the **(a)** milk yield, **(b)** fat yield, **(c)** fat content, **(d)** protein, **(e)** protein content, **(f)** combined fat and protein (CFP), **(g)** productive life (PL), **(h)** somatic cell score (SCS), **(i)** daughter pregnancy rate (DPR), **(j)** livability (LV), **(k)** udder composite (UDC), **(l)** feet–legs composite (FLC) **(m)** total performance index (TPI), and **(n)** net merit (NM).

In this study, we genomically tested 805 purebred Holstein Friesian cows and evaluated the genetic merit of the animals based on the *CSN2* and *CSN3* genotypes. We observed adequate variation and genetic diversity levels in both markers (Table 1). Furthermore, the *CSN2* and *CSN3* markers exhibited intermediate and high PIC levels, respectively. Comparison of the genomic merit values based on the genotypes (Figs. 1, 2) revealed significant differences (Tables 2, 3). Concerning the *CSN*2 g.8101C > A (p.His67Pro) alteration, A2A2 animals had higher PTA milk values than the A1A1 animals and heterozygotes. However, this difference was not substantiated in the ANOVA ( P=0.075 ). Previous studies have revealed varying results with respect to the effects of *CSN2* g.8101C > A (p.His67Pro) genotypes on milk yield and content. Many researchers have reported that the A2 allele does not cause any deterioration in milk production and even positively affects milk yield. However, a considerable number of studies have pointed out a negative relationship between the A2A2 genotype and reproductive traits. Morris et al. (2005) indicated that A2A2 cows have a significantly higher advantage regarding the milk value per day (2.1 %) than those with A1A1 and A1A2 genotypes based on the results of combining the milk yield data and the protein and fat percentage. Nilsen et al. (2009) reported a tendency towards increased milk and protein yields for haplotypes containing the *CSN2*-A2 variant. In a study by Soyudal et al. (2019), the A2A2 genotype was associated with the desired results for 305 d milk yield, days before peak milk production, and protein yield. Miluchová et al. (2023) found that the A2 allele positively influenced the protein in kilograms and, partially, the protein content. Similarly, Ivanković et al. (2021) reported that higher milk production was determined in A2A2-genotype animals for conventional breeds. It is important to note that Soyudal et al. (2019), Ivanković et al. (2021), and Miluchová et al. (2023) observed an increased milk fat percentage in A1A1 animals compared with those with the A2A2 genotype. While evaluating this situation, of course, the negative correlation between milk yield and fat or protein content should be considered. The g.8101C > A alteration in bovine *CSN2* may also change the biochemical properties of the milk. De Vitte et al. (2022) showed an association of the *CSN2* variants with the amino–fatty acid composition and milk color. These researchers pointed out that A2A2 milk was significantly higher in polyunsaturated fatty acids, omega-3, and omega-6, whereas it was lower with respect to the saturated fatty acid content in milk fats. Heck et al. (2009) observed that cows with the A1 allele had a lower protein yield than cows with the A2 allele, resulting from decreased milk production. Oleński et al. (2012) reported that the A2 allele increases breeding values for milk and milk protein yields. Conversely, Kučerová et al. (2006) indicated a significant association between the A1A1 genotype and the highest breeding value for milk yield. It is important to note that differences in the breed of cows, population size, methods of expressing phenotypic traits, genotype frequencies, genotypic interactions, and the power of statistical models may influence the results remarkably. In this study, the A2A2 genotype was significantly associated with better genetic merit for milk protein, CFP, and PL (Fig. 1). Cows with this genotype also had the highest TPI and NM values (Table 2). NM measures the additional net profit that an offspring of an animal will provide over its lifetime. Income and expenses for a typical dairy operation have been estimated to calculate overall net profit (Holstein Association USA, 2024). Many articles have reported that A2 conversion is more profitable for dairy farms (Cieślińska et al., 2022; Kearney et al., 2005; Oleński et al., 2012). Indeed, our results corroborate this suggestion regarding the breeding values of milk production. In recent dairy breeding, profitability can only be achieved by performing genetic tests on animals belonging to *CSN2* genotypes, determining genetic merits for various traits (including health and reproductive performance), and evaluating GEBVs.

Along with *CSN2*, one of the essential casein genes is *CSN3* in dairy cattle. Kučerová et al. (2006) reported that differences between the genotypes at the *CSN3* locus regarding breeding values for protein content and protein yield were significant. These researchers indicated that the AA genotype was associated with a low average breeding value for milk yield. Moreover, the BE genotype was related to the highest breeding value for protein yield but to a lower value for protein content. This study partially confirmed their suggestions. However, we observed that the BE haplotype exhibits significantly higher breeding values for protein content and yields ( P<0.01 ) than those with alternative haplotypes (Fig. 2d, e). The animals with this haplotype also have the highest NM value (Table 3). Further, we found that BE is associated with the highest breeding values for the CFP but the lowest values for the SCS and DPR. Kaminski et al. (2002) indicated that the highest breeding value for milk yield and the lowest for protein content were related to the AA genotype. Kučerová et al. (2005) reported that higher breeding values for protein content but lower values for protein yield were associated with the BB genotype. Similarly, we observed that BB is associated with a significant increase in the breeding value for protein content. Here, it is worth noting that we also found that BB-haplotype animals exhibit the highest PTAs for PL ( P<0.01 ). The current information on the association between the breeding values and the *CSN3* haplotypes is relatively low, especially compared with the bovine *CSN2* gene. This study may provide important clues with respect to evaluating the genetic merit of animals carrying different *CSN3* haplotypes.

Another critical issue, besides milk production, that needs to be evaluated on a herd basis is health and reproductive traits. The literature shows that studies examining the effects of the A2 allele have focused on milk production characteristics and that information on reproductive performance is limited. Indeed, this assertion holds validity with regard to other milk proteins as well (Czerniawska-Piatkowska et al., 2023). Ardicli et al. (2019a) reported that the A2A2 genotype had higher mean values for days before the first insemination and the pregnancy interval. The effects of *CSN2* genotypes, regarding g.8101C > A (p.His67Pro) alteration, on reproductive performance should be monitored on a herd basis, and GEBVs should be considered in this respect. Genomic test reports consist of PTAs for several traits to compare the breeder's genetic merit. Breeder selection decisions by commercial dairy farmers play a pivotal role in achieving high profit and sustainability. Genomic testing of both cows and sires reveals reliable data for an innovative breeding scheme. Unfortunately, the best breeding schemes are affected by volatile prices (De Vries, 2017). Although genetic testing results in an extra cost for the farm, genomic testing is currently invaluable with respect to profitability from a forward-looking perspective. On the other hand, cattle export worldwide is a huge market, and genetic testing is crucial for selecting animals to be imported (Ardicli et al., 2019b). Genomic testing of females on the farm can be profitable, depending on the fraction of surplus heifers that can be created and smart breeding decisions regarding sexed semen (De Vries, 2017). Kearney et al. (2005) indicated that the benefits of using homozygous sires would be most significant for those herds that already have a high frequency of the favorable allele in countries where a homozygous genotype is commercially advantageous (e.g., A2A2 in New Zealand). Commercialized A2 milk can be marketed at higher prices; however, it is still essential to note that monitoring the breeder candidates based on the genetic merit data of other crucial traits, such as SCS, DPR, PL, UDC, and FLC, allows for an accurate selection of dairy cattle. Some other indexes are also increasing with respect to their importance in genetic evaluation, such as the genetic merit for stillbirth (Holstein Association USA, 2024).

In this study, the highest SCS value belongs to the heterozygous genotype ( P<0.05 ) in the *CSN2* marker. Notably, the A1A1-genotype-carrier animals have the highest UDC score ( P<0.05 ); however, the UDC remained positive for all three genotypes. Although these two critical parameters lag far behind milk yield for many years, current studies have reveal that a healthy udder structure is much more important than the yield at the herd level. In the present study, we also observed that the *CSN3*-AA haplotype has the highest SCS ( P<0.001 ). Nevertheless, as in the *CSN2* gene, the mean values of the PTA of UDC remained positive for all six haplotypes (Table 3). The SCS and UDC indicate genetic susceptibility for udder health. Using the PTA of SCS in an index is recommended so that appropriate selection might be given to improving mastitis resistance. Genetic selection to reduce SCS does not replace superior management and preventative care as the most effective means of controlling mastitis.

Using the PTA of SCS in an index and placing 5 % as much weight on SCS as on yield will help slow the increase in mastitis without sacrificing much in terms of increased yield (Holstein Association USA, 2024). The popularity of A2 milk worldwide is increasing day by day. However, the biological effects of BCM7 (from A1 milk) on human health remain uncertain, and more scientific evidence of these effects is required (Summer et al., 2020). If the health risks of *CSN2*-A1 milk consumption are confirmed, consumers may wish to reduce their intake or remove this kind of milk from their diet. Thus, farmers should take the appropriate steps to allow for a systematic reduction in the number of cows and bulls with the A1 allele of *CSN2* and, consequently, reduce the spread of this undesirable allele in a dairy cattle population (Cieślińska et al., 2022). At this stage, the herd's breeding values regarding important health and reproductive characteristics should be monitored, and other relevant gene regions, such as the *CSN3*, should be considered. Breeders who wish to produce A2 milk should adopt this aspect in all essential steps, such as replacing heifers in the herd, culling the animals carrying A1 alleles, and using A2 semen. A selection scheme created without good planning and focusing on a single genomic variant can lead to irreversible negative affects on herd genetics.

In conclusion, this report focused on genetic variation in the *CSN2* and *CSN3* genes and their relationships with genomic merit in a relatively sizable Holstein Friesian cattle population. Among the studied genotypes and haplotypes, statistically significant associations were observed for both production and health/reproductive traits. The A2A2 genotype and the BE haplotype exhibited the highest net merit. However, it is important to note that the animals with these genotypes had the lowest predicted transmitting abilities for some reproductive or health traits, such as the udder composite and daughter pregnancy rate traits. The analysis in this work has confirmed the findings of some previously published papers and also presents some novel associations. Furthermore, the present paper demonstrates how critical a complex and detailed evaluation is in selecting the breeder cows based on a particular genotypic variant. This assessment may be helpful for further analyses regarding selection decisions involving the breeding values of the candidate animals.

## Supplement

10.5194/aab-67-61-2024-supplement The supplement related to this article is available online at: https://doi.org/10.5194/aab-67-61-2024-supplement.

## Supplement

10.5194/aab-67-61-2024-supplement
10.5194/aab-67-61-2024-supplement The supplement related to this article is available online at: https://doi.org/10.5194/aab-67-61-2024-supplement.

## Data Availability

The datasets generated are available from the corresponding author on request.

## Appendix

The supplement related to this article is available online at: https://doi.org/10.5194/aab-67-61-2024-supplement.

## References

[bib1.bib1] Ardicli S, Samli H, Soyudal B, Dincel D, Balci F (2019). Evaluation of candidate gene effects and environmental factors on reproductive performance of Holstein cows. S Afr J Anim Sci.

[bib1.bib2] Ardicli S, Ustuner H, Arslan O, Kandazoglu O (2019). Variability of CAPN1 g. 5709 C 
>
 G and MYF5 g. 1911 A 
>
 G Polymorphisms in Beef Cattle Imported from Brazil to Turkey. Livestock Studies.

[bib1.bib3] Ardicli S, Samli H, Balci F (2023). Analysis of bovine beta-casein A1 and A2 allele frequency in Holstein-Friesian cows by Real-time PCR with fluorescent hybridization probes. Vet Arhiv.

[bib1.bib4] Bell SJ, Grochoski GT, Clarke AJ (2006). Health implications of milk containing 
β
-casein with the A2 genetic variant. Crit Rev Food Sci.

[bib1.bib5] Botstein D, White RL, Skolnick M, Davis RW (1980). Construction of a genetic linkage map in man using restriction fragment length polymorphisms. Am J Hum Genet.

[bib1.bib6] Cieślińska A, Fiedorowicz E, Rozmus D, Sienkiewicz-Szłapka E, Jarmołowska B, Kamiński S (2022). Does a Little Difference Make a Big Difference? Bovine 
β
-Casein A1 and A2 Variants and Human Health – An Update. Int J Mol Sci.

[bib1.bib7] Czerniawska-Piatkowska E, Cioch-Szklarz B, Kowalczyk A, Wrzecinska M, Wójcik J, Kordan W, Araujo JP, Cerqueira JL, Kossakowski K, Cwynar P, Sablik P (2023). Relationship between milk protein polymorphism and selected cows' reproductive indices. Animals.

[bib1.bib8] De Koning D-J (2006). Conflicting candidates for cattle QTLs. Trends Genet.

[bib1.bib9] De Vitte K, Kerziene S, Klementavičiūtė J, De Vitte M, Mišeikienė R, Kudlinskienė I, Čepaitė J, Dilbiene V, Stankevičius R (2022). Relationship of 
β
-casein genotypes (A1A1, A1A2 and A2A2) to the physicochemical composition and sensory characteristics of cows' milk. J Appl Anim Res.

[bib1.bib10] De Vries A (2017). Innovative Breeding Schemes: Best Combinations of Genomics, Semen Type, and Culling. 2017 Western Dairy Management Conference Proceedings.

[bib1.bib11] Falconer DS, Mackay TFC (1996). Introduction to Quantitative Genetics.

[bib1.bib12] Giglioti R, Gutmanis G, Katiki LM, Okino CH, de Sena Oliveira MC, Vercesi Filho AE (2020). New high-sensitive rhAmp method for A1 allele detection in A2 milk samples. Food Chem.

[bib1.bib13] Goddard M, Hayes B (2007). Genomic selection. J Anim Breed Genet.

[bib1.bib14] Hayes B, Goddard M (2010). Genome-wide association and genomic selection in animal breeding. Genome.

[bib1.bib15] Heck J, Schennink A, Van Valenberg H, Bovenhuis H, Visker M, Van Arendonk J, Van Hooijdonk A (2009). Effects of milk protein variants on the protein composition of bovine milk. J Dairy Sci.

[bib1.bib16] Holstein Association USA.

[bib1.bib17] Ivanković A, Pećina M, Ramljak J, Pašić V (2021). Genetic polymorphism and effect on milk production of CSN2 gene in conventional and local cattle breeds in Croatia. Mljekarstvo.

[bib1.bib18] Jiménez-Montenegro L, Alfonso L, Mendizabal JA, Urrutia O (2022). Worldwide Research Trends on Milk Containing Only A2 
β
-Casein: A Bibliometric Study. Animals.

[bib1.bib19] Kaminski S, Rymkiewicz-Schymczyk J, Wojcik E, Rusc A (2002). Associations between bovine milk protein genotypes and haplotypes and the breeding value of Polish Black-and-White bulls. J Anim Feed Sci.

[bib1.bib20] Kearney J, Amer P, Villanueva B (2005). Cumulative discounted expressions of sire genotypes for the complex vertebral malformation and 
β
-casein loci in commercial dairy herds. J Dairy Sci.

[bib1.bib21] Kučerová J, Matějíček A, Jandurová O, Sorensen P, Němcová E, Štípková M, Kott T, Bouška J, Frelich J (2006). Milk protein genes CSN1S1, CSN2, CSN3, LGB and their relation to genetic values of milk production parameters in Czech Fleckvieh. Czech J Anim Sci.

[bib1.bib22] Kučerová J, Němcová E, Štípková M, Jandurová O, Matějíček A, Bouška J (2005). The association between CSN3 genotypes and milk production parameters in Czech Pied Cattle.

[bib1.bib23] Louis EJ, Dempster ER (1987). An exact test for Hardy-Weinberg and multiple alleles. Biometrics.

[bib1.bib24] Mencarini I (2013). A simulation model of dairy herd conversion to produce A2 milk [master's thesis].

[bib1.bib25] Meuwissen T, Hayes B, Goddard M (2016). Genomic selection: A paradigm shift in animal breeding. Anim Front.

[bib1.bib26] Miluchová M, Gábor M, Candrák J (2023). The Effect of the Genotypes of the CSN2 Gene on Test-Day Milk Yields in the Slovak Holstein Cow. Agriculture.

[bib1.bib27] Morris C, Hickey S, Cullen N, Prosser C, Anderson R, Tate M (2005). Associations between 
β
-casein genotype and milk yield and composition in grazing dairy cows. New Zealand J Agric Res.

[bib1.bib28] Nei M, Roychoudhury A (1974). Sampling variances of heterozygosity and genetic distance. Genetics.

[bib1.bib29] Nilsen H, Olsen HG, Hayes B, Sehested E, Svendsen M, Nome T, Meuwissen T, Lien S (2009). Casein haplotypes and their association with milk production traits in Norwegian Red cattle. Genet Sel Evol.

[bib1.bib30] National Research Council (NRC) (2001). Nutrient Requirements of Dairy Cattle: 7th Revised Edition.

[bib1.bib31] Oleński K, Cieślińska A, Suchocki T, Szyda J, Kamiński S (2012). Polymorphism in coding and regulatory sequences of beta-casein gene is associated with milk production traits in Holstein-Friesian cattle. Anim Sci Pap Rep.

[bib1.bib32] Prasad A, Kothari N (2022). Cow products: boon to human health and food security. Trop Anim Health Pro.

[bib1.bib33] Rector K (2009). Selecting, Marketing and Rebuilding a Herd of Genetically Superior Animals.

[bib1.bib34] Rodriguez-Zas S, Southey B, Heyen D, Lewin H (2002). Interval and composite interval mapping of somatic cell score, yield, and components of milk in dairy cattle. J Dairy Sci.

[bib1.bib35] Soyudal B, Ardıçlı S, Şamlı H, Dinçel D, Balcı F (2019). Association of polymorphisms in the CSN2, CSN3, LGB and LALBA genes with milk production traits in Holstein cows raised in Turkey. J Hell Vet Med Soc.

[bib1.bib36] Summer A, Di Frangia F, Ajmone Marsan P, De Noni I, Malacarne M (2020). Occurrence, biological properties and potential effects on human health of 
β
-casomorphin 7: Current knowledge and concerns. Crit Rev Food Sci.

[bib1.bib37] Livestock Statistics.

[bib1.bib38] Wright J, VanRaden P (2016). Genetic evaluation of dairy cow livability. J Anim Sci.

