# Supplementary material for: Associations of bovine beta-casein and kappa-casein genotypes with genomic merit in Holstein Friesian cattle
Source: Arch Anim Breed. 2024 Feb 14;67(1):61–71. doi: 10.5194/aab-67-61-2024 (PMC12344668; doi:10.5194/aab-67-61-2024)
Supplement: The supplement related to this article is available online at: https://doi.org/10.5194/aab-67-61-2024-supplement. [file aab-67-61-2024-supplement.pdf]

Supplement of Arch. Anim. Breed., 67, 61–71, 2024
https://doi.org/10.5194/aab-67-61-2024-supplement
© Author(s) 2024. CC BY 4.0 License.

Open Access
Archives Animal Breeding

Supplement of

Associations of bovine beta-casein and kappa-casein genotypes with genomic merit in Holstein Friesian cattle

Sena Ardicli et al.

Correspondence to: Sena Ardicli (sardicli@uludag.edu.tr)

The copyright of individual parts of the supplement might differ from the article licence.

Table S1. The descriptive data on genetic merit values (PTAs, TPI, and NM) of the studied Holstein-Friesian population (*n*=805).

Variable	Mean	Standard Error	Coefficient of Variation	Minimum	Maximum
Milk yield	708.20	19.500	78.90	-1948.00	2144.00
Fat	33.61	0.726	61.79	-33.00	105.00
% Fat	0.02	0.003	385.13	-0.21	0.29
Protein	26.18	0.478	52.18	-39.00	62.00
% Protein	0.01	0.001	255.14	-0.10	0.10
CFP	59.79	1.080	51.78	-72.00	153.00
PL	2.84	0.052	51.98	-1.60	6.60
SCS	2.91	0.004	4.07	2.51	3.31
DPR	-0.08	0.048	-1705.19	-4.40	4.30
LV	0.57	0.055	253.18	-3.50	5.40
UDC	0.33	0.031	254.29	-3.00	3.60
FLC	0.09	0.021	616.92	-2.61	1.74
TPI	2376.30	6.030	7.08	1859.00	2884.00
NM	413.53	6.050	41.83	-190.00	904.00

CFP: Combined fat and protein; PL: Productive life; SCS: Somatic cell score; DPR: Daughter pregnancy rate; LV: Livability; UDC: Udder Composite; FLC: feet-legs composite; TPI: Total performance index; NM: Net merit
